# Management lacrimal sac abscesses using lacrimal probe and crawford silicon tube

**DOI:** 10.1186/s12886-016-0378-y

**Published:** 2016-11-30

**Authors:** Lin Lin, Li Yang, Xiuming Jin, Yingying Zhao, Fangli Fan

**Affiliations:** 1 Eye Center, Second Affiliated Hospital of Zhejiang University School of Medicine, No. 88 Jiefang Rd, Hangzhou, 310009 China; 2 The Second Affiliated Hospital of Zhejiang Chinese Medical University, No. 318 Chaowang Rd, Hangzhou, 310005 China

**Keywords:** Lacrimal sac abscesses, Lacrimal intubation, Crawford tube, Acute dacryocystitis

## Abstract

**Background:**

Treatment of lacrimal sac abscess of the traditional surgical approach may result in complications from cutaneous fistula formation, damage the sac, cause skin scarring and even have the potential for inducing cicatricial ectropion. We designed a new treatment scheme that is expected to achieve internal drainage with the use of lacrimal probe and crawford silicon tube.

**Methods:**

A prospective study was performed for the management of lacrimal sac abscesses. All suitable patients from January 2011 to June 2014 were managed by lacrimal probe and crawford tube insertion. Postoperatively, patients received 0.5% Levofloxacin eye drops four times per day and oral Levofloxacin tablets 0.5 g once per day for four days. Follow-up times were for more than three months after removing the Crawford tube. The condition of the lacrimal sac and the patient’s symptoms were carefully evaluated.

**Results:**

Fourteen patients suffering from lacrimal sac abscesses were included in this study. A history of chronic dacryocystitis was found in six patients, after acute dacryocystitis was found in three patients, and nasolacrimal occlusion with epiphora was found in other five patients. Resolution of signs and symptoms of lacrimal sac abscesses in all fourteen patients. No recurrence of lacrimal sac abscesses occurred during the median follow up period of 20.9 ± 7.8 months (range 6–36 months). Epiphora reoccurred in four patients.

**Conclusions:**

Lacrimal probe and crawford silicon tube is successful as a procedure of choice for lacrimal sac abscesses. The insertion of a Crawford tube also offers potential advantages over standard treatment with the lack of recurrence of dacryocystitis or infection in post-surgical patients.

## Background

Lacrimal sac abscesses usually occurs because of an obstruction of the nasolacrimal duct or after acute dacryocystitis [1]. Because of distal nasolacrimal duct obstruction (NLDO), abscess usually creates an abscess within the sac [2, 3]. The conventional treatment of lacrimal sac abscess formation includes the use of systemic antibiotics, percutaneous drainage of the abscess, and external dacryocystorhinostomy (DCR) [2]. The traditional therapy, however, may result in complications from cutaneous fistula formation, damage the sac, cause skin scarring affecting appearance, and even have the potential for inducing cicatricial ectropion [4]. Other risks include recurrent infections before DCR can be performed and prolonged infection due to poor antibiotic abscess penetration. External DCR can result in cutaneous scar formation and may also disrupt medial canthal anatomy [5, 6]. Primary endonasal DCR offers potential advantages over standard treatment [7, 8]. However, endonasal DCR can result in a significant hemorrhage during incisions of the lateral mucosa to create an ostium and drain the abscess [9].

The aim of this study was to prospectively evaluate the use of Crawford tubes in the primary treatment of acute dacryocystitis with abscess formation.

## Methods

Patients presenting to the Eye Center, Affiliated Second Hospital, School of Medicine, Zhejiang University, with lacrimal sac abscess between January 2010 and June 2014 were selected as possible candidates for this study. The diagnosis of lacrimal sac abscess was based on the clinical history (such as chronic dacryocystitis or after acute dacryocystitis) and the sense of volatility when pressing lacrimal sac. Patients were assessed jointly by an ophthalmologist and an otolaryngologist for their suitability for primary internal drainage via lacrimal probe and crawford silicon tube insertion approach. Preoperative assessments included a complete eye and intranasal examination. Exclusion criteria included bloodstained tears or a hard mass suggesting a possible lacrimal sac neoplasia, some nasal abnormalities and patients unwilling to accept this approach. This was an option of care decided upon the clinician according to the patient's condition and obtained consent of the patient. All suitable patients were initially managed with oral Levofloxacin tablets (Daiichi Pharmaceutical Co, Ltd) 0.5 g once per day for three days and informed consent was obtained before surgery. This study was approved by the Ethics Committee of the Second Affiliated Hospital of Zhejiang University School of Medicine of China and complied with the tenets of the Declaration of Helsinki.

A single experienced specialist lacrimal surgeon performed all surgical procedures. Elderly patients or patients have a high painless requirements choose general anesthesia. The surgical procedures were previously described [10]. First, the inferior nasal meatus was treated with a pledget soaked in 0.4% Oxybuprocaine Hydrochloride (Santen Pharmaceutical Co, Ltd) and 1% ephedrine hydrochloride solution. Then a dilator was used to dilate the lacrimal puncta. After dilation of the puncta, an attempt was made to probe the nasolacrimal duct through both the upper and lower puncta. A Crawford silicone tube (Bausch & Lomb Freda) was passed from both the lower and upper punctum to the nasolacrimal duct and pulled out of the nose. The two ends were tied beside the nose, excess tubing was cut off, and the endpoint was left in the inferior nasal meatus for at least three months. During the surgery, the surgeon try to follow the anatomy of lacrimal duct, avoid blind force, this can effectively avoid the formation of false track.

Postoperatively, the patients received 0.5% Levofloxacin eye drops (Santen Pharmaceutical Co, Ltd) four times per day and oral Levofloxacin tablets (Daiichi Pharmaceutical Co, Ltd) 0.5 g once per day for four days. Follow-up evaluations occurred at day one, week one, month one, month three and month six. Follow-up examinations occurred for longer than six months after removing the Crawford tube.

## Results

**Fig. 1:**
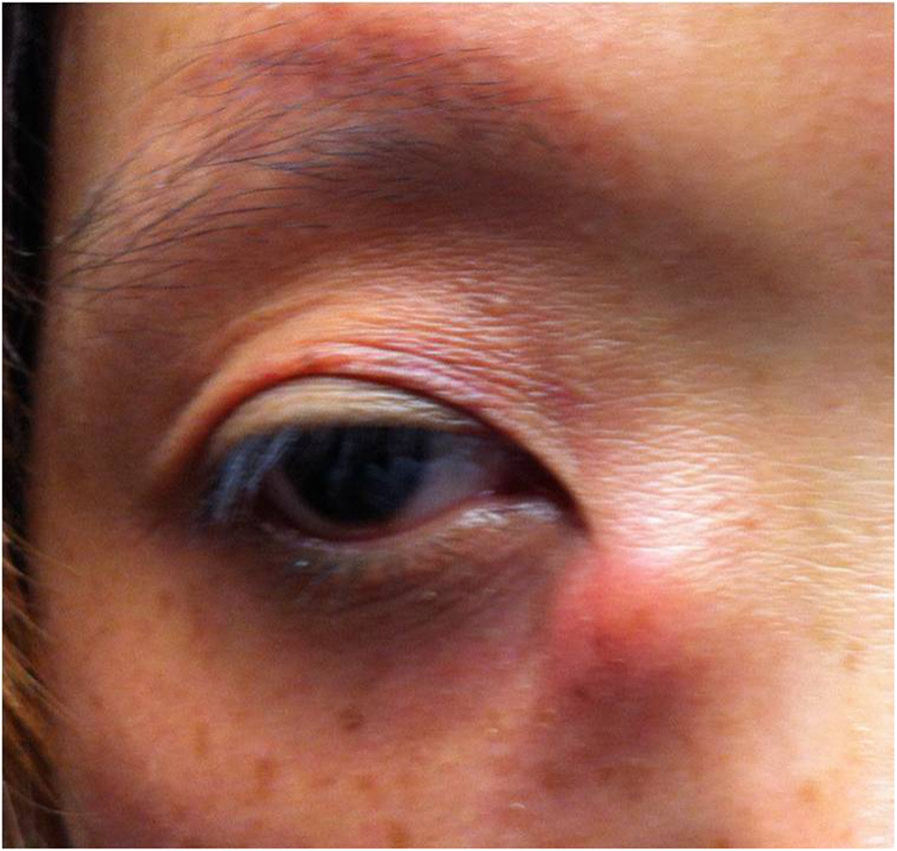
Photograph showing lacrimal sac abscesses

Fourteen patients were studied (mean age: 53.7 ± 8.8 years, range: 39–68 years; three men and eleven women). Twelve of them accepted localized anesthesia, while the other two accepted general anesthesia. The duration of surgical procedures were about ten to fifteen minutes. During operation, VAS pain scores of the patients were generally less than 3 points. Included patients’ details are summarized in Table 1. All patients had monocular dacryocystitis involvement. All patients had swelling with dynamic abscess in the lacrimal sac (Fig. 1). There is no tenderness around lacrimal sac abscess. A history of chronic dacryocystitis was found in six patients, after acute dacryocystitis was found in three patients, and nasolacrimal occlusion with epiphora was found in other five patients. No patients had a history of previous nasolacrimal surgery. One patient suffered punctum dehiscence and one patient had a medial canthal cutaneous fistula on presentation.

**Table 1:** Patient profile of acute dacryocystitis complicated by abscess formation

Patient No.	Sex	Age group^a^	History of chronic dacryocystitis	Anesthesia	Course of abscess (days)	Crawford tube placement duration (months)	Follow up (months)
1	F	5	yes	local	10	3	30
2	F	4	no	local	7	5	24
3	M	5	yes	local	9	3.5	21
4	F	6	no	general	13	6	26
5	F	6	yes	local	21	4.2	21
6	M	3	no	local	10	7	24
7	F	5	yes	local	8	6	36
8	F	4	no	local	6	3	20
9	F	5	no	local	15	5.5	27
10	F	5	yes	general	14	4	18
11	F	6	no	local	7	5.5	14
12	F	4	no	local	6	6	14
13	F	5	no	local	8	5.5	12
14	M	5	yes	local	12	4	6

^a^Age group: 3: ≥30 and <40, 4: ≥40 and <50, 5: ≥50 and <60

**Fig. 2:**
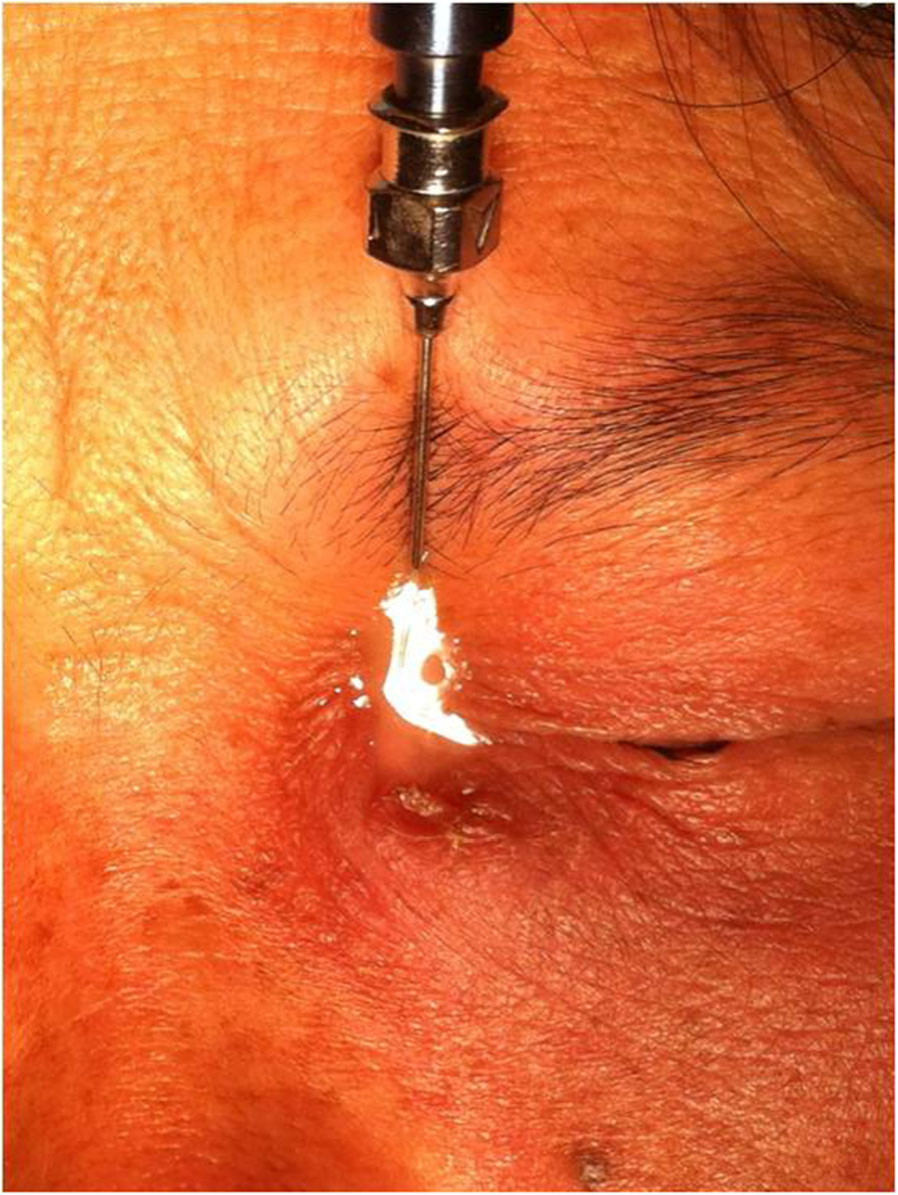
The photograph shows the lacrimal probe. When we use the lacrimal needle inserted into the lacrimal duct, a lot of pus will spill from the punctum. After surgery, Crawford tube may maintain the drainage and eliminate the abscess cavity

**Fig. 3:**
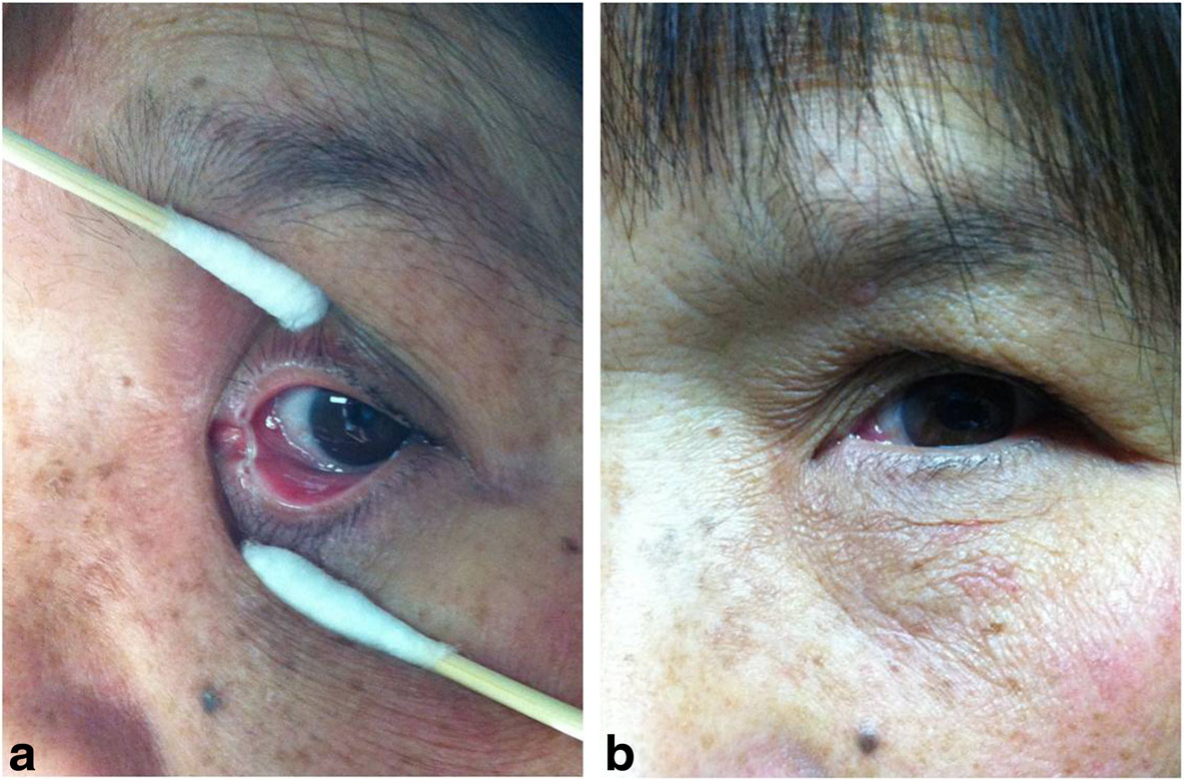
Resolution of signs and symptoms of acute dacryocystitis: one month (Fig. 3**a**) and three months (Fig. 3**b**) after lacrimal intubation

**Fig. 4:**
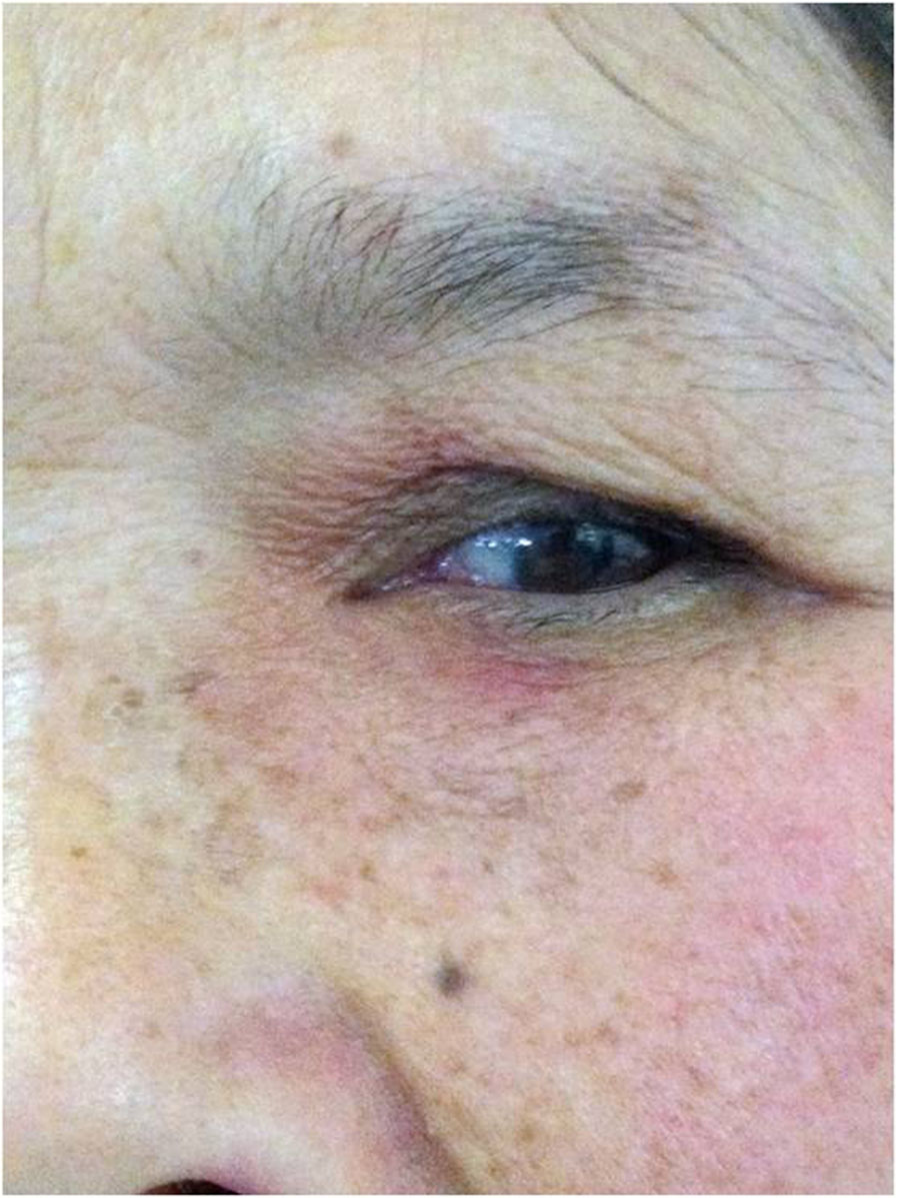
Photograph showing outward appearance three months after removing the Crawford tube

Resolution of the signs and symptoms of lacrimal sac abscess in all fourteen patients. The abscesses were successfully drained after probing in each patient (Fig. 2). No recurrence of lacrimal sac abscesses occurred during the follow up period. Epiphora reoccurred in four patients. The Crawford tube remained in place for more than three months after the operation (mean: 4.9 ± 1.3 months) as shown in Fig. 3. The mean total follow-up time was 20.9 ± 7.8 months (range 6–36 months) after pulling out the Crawford tube (Fig. 4). During the final follow-up, epiphora recurred in four patients and these patients may need further dacryocystorhinostomy while the other ten patients didn’t show any symptom of epiphora and the irrigation was performed to confirm anatomic patency. One patient suffered the lacrimal sac fistula due to long-term abscesses and pus outbreaks from the skin before the surgery. The end result of this patient is healed with scar after intubation. Another patient developed an abscess with the Crawford tube one week after the surgery. After a gentle massage, the abscess disappeared and did not return.

## Discussion

The challenges in treating lacrimal sac abscess are well known. Surgical intervention by means of incision and drainage (I&D), has been the gold standard of treatment. However, skin incisions can cause scarring and have the potential for inducing cicatricial ectropion. These modalities may also lead to persistent lacrimal sac-cutaneous fistulas [11]. More troublesome, is after I&D, a definitive treatment, such as DCR, is required. Unfortunately, these patients have to go through the pain of surgery twice. The external scars seen in I&D are absent, making it a cosmetically superior approach, and also functionally better, as the lacrimal pump is not damaged since the orbicularis oculi muscle is not incised [12].

Basic treatment principles for lacrimal sac abscess may include controlling infections, maintaining the drainage of the lacrimal sac, and eliminating the abscess cavity. The thought of treating lacrimal sac abscess by proceeding lacrimal duct intubation came from a patient. The patient insisted not to drainage by making an incision on the abscess. Under the circumstances, we consulted many of the related literatures about treating lacrimal sac abscess and finally found that it could be cured by performing dacryocystorhinostomy to realize internal drainage under the nasal endoscope. So we finally designed a scheme of treating acute dacryocystitis with canalicular intubation to realize internal drainage. We probed lacrimal to drainage the lacrimal sac abscess, performed Crawford tube insertions to maintain the drainage and eliminate the abscess cavity; this, combined with topical and oral antibiotic therapies to control infections, yielded excellent results. Our results show a high success rate of Crawford tube insertion for the treatment of lacrimal sac abscess with all patients demonstrating a significant improvement in signs and symptoms. Simple incision and drainage without intubation has shown a success rate of 91.7–96.2% in several studies with a follow-up of more than two years [4, 13]. However the patients accepted simple incision and drainage may need further external or endonasal DCR to manage the epiphora. Our results shown anatomic patency in ten patients and suggested at least 71.4% (10/14) of patients avoided the skin scaring and also did not need further surgery. Endonasal DCR with silicon tube stents was recommended as the better treatment of choice for acute dacryocystitis with lacrimal abscesses [14, 15]. However endonasal DCR cannot be performed in many hospitals especially in China. The simply insertion of a Crawford tube may be a choice for acute lacrimal abscesses. This treatment solves two important problems at the same time: first, it drains the abscess, and second, it eliminates the NLDO. This method has the advantage of eliminating the risk of scarring and surgery.

One patient suffered punctum dehiscence. This might be related to the injury to the dacryon caused by the operations during which we performed expanding on the lacrimal passage, washing, and cannula together with the postoperative catheter pull. One patient suffered skin scarring because of long-term abscesses. Another patient developed an abscess with the Crawford tube one week after the surgery. After a gentle massage, the abscess disappeared and did not return. The reason of the returning of the abscess could be that the cannula might make a very limited damage onto the vesicle wall of the abscess after which the wall unioned gradually and slowly to form a new sac. But the weak points would fracture after massaging, thus resulting the disappearing of the abscess. Lacrimal sac abscesses close to the front wall may cause poor drainage, and may be the most likely reason for this complication. No other complications, such as persistent corneal erosion, bacterial keratitis, or lid infection, were found in our study.

The insertion of a Crawford tube is quick, relatively painless, non-invasive and generally without significant risk to the patient. The rationale is to widen the canaliculus and nasolacrimal duct and place a silicone tube inside to prevent subsequent narrowing, similar to how angioplasty is used to widen arteries. The lacrimal probe and insertion of a Crawford tube also offers potential advantages over standard treatment with rapid improvement in pain, earlier resolution of infection, and the economic benefits of reduced patient length of stay without the need for later readmission for external or endonasal DCR. However there are a number of limitations to this study, which include this is a small sample size, non-comparative study, lack of microbiological culture and imaging used. A large-scale prospective trial, with longer follow-up, seems warranted to more clearly define its role in treating lacrimal sac abscesses.

## Conclusions

Lacrimal probe and crawford silicon tube is successful as a procedure of choice for lacrimal sac abscesses. The insertion of a Crawford tube also offers potential advantages over standard treatment with the lack of recurrence of dacryocystitis or infection in post-surgical patients.
